# Automated Lunar Crater Identification with Chandrayaan-2 TMC-2 Images using Deep Convolutional Neural Networks

**DOI:** 10.1038/s41598-024-58438-4

**Published:** 2024-04-08

**Authors:** Mimansa Sinha, Sanchita Paul, Mili Ghosh, Sachi Nandan Mohanty, Radha Mohan Pattanayak

**Affiliations:** 1 Department of Computer Science and Engineering, Birla Institute of Technology, Mesra, Ranchi, India; 2 Department of Remote Sensing, Birla Institute of Technology, Mesra, Ranchi, India; 3 School of Computer Science and Engineering (SCOPE), VIT-AP University, Amaravati, Andhra Pradesh India

**Keywords:** Deep learning, ResNet, U-Net, FCNN, TMC-2, Chandrayaan-2, Canny edge detection, Endocrinology, Gastroenterology, Energy science and technology, Engineering

## Abstract

Terrestrial planets and their moons have impact craters, contributing significantly to the complex geomorphology of planetary bodies in our Solar System. Traditional crater identification methods struggle with accuracy because of the diverse forms, locations, and sizes of the craters. Our main aim is to locate lunar craters using images from Terrain Mapping Camera-2 (TMC-2) onboard the Chandrayaan-II satellite. The crater-based U-Net model, a convolutional neural network frequently used in image segmentation tasks, is a deep learning method presented in this study. The task of crater detection was accomplished with the proposed model in two steps: initially, it was trained using Resnet18 as the backbone and U-Net based on Image Net as weights. Secondly, TMC-2 images from Chandrayaan-2 were used to detect craters based on the trained model. The model proposed in this study comprises a neural network, feature extractor, and optimization technique for lunar crater detection. The model achieves 80.95% accuracy using unannotated data and precision and recall are much better with annotated data with an accuracy of 86.91% in object detection with TMC-2 ortho images. 2000 images have been considered for the present work as manual annotation is a time-consuming process and the inclusion of more images can enhance the performance score of the model proposed.

## Introduction

Numerous lunar missions, such as Apollo, Clementine, Chandrayaan-1, Selenological and Engineering Explore (SELENE), Chang'E, Lunar Crater Observation and Sensing Satellite (LCROSS)/Lunar Reconnaissance Orbiter (LRO) and Chandrayaan-2 have successfully gathered important data over several decades^1,2^. More than 109,000 new craters have been found on the Moon’s low- and mid-latitude regions using Artificial Intelligence (AI) and data gathered by Chinese lunar orbiters^3^. Understanding the history and evolution of the Moon, as well as finding suitable landing locations for future missions, requires the study of lunar craters. The amount of data contained in these images grows quicker than the capacity of human operators to examine and gather the necessary information from them to describe the planetary body in question. Impact crater density, patterns, and forms are important to study to comprehend a planet's geological past. Simple craters, intricate craters, and multi-ring basins are the three basic crater types. Impact craters are classified as simple or complex craters based on their structural and morphological properties. Simple craters are depressions with polygonal to circular bowl-shaped rims that are blanketed with continuous ejects^4^. A central uplift, an inward collapse of single, several, or continuous blocks over the rim, and a flat or humpbacked floor are just a few internal features that may be utilized to identify complex craters^5^. Transitional craters are more commonly found in highland terrains having abrupt slope increases. These craters have localized slumps and terraces^6^. In terms of strength and topography, the terrain is more likely to facilitate the occurrence of simple craters, and complex craters are more likely to occur if there is variability in these elements, such as differences in strength, terrain, or lithology. It is important to remember that the creation of impact craters is a complicated process that depends on several variables, including the impactor's size and velocity, the target material's composition, and the impact angle^7^. On the moon, corrosion and water weathering are much weaker than they are on Earth, therefore impact craters, even those formed 4 billion years ago, are frequently still intact^8^. Although, Lunar crater morphology degrades slowly by the formation of new impacts and their ejecta, not significantly by weathering and tectonism^9^. We can learn more about the early Earth's composition by studying the unchanging lunar surface, and this knowledge can help us find evidence buried behind the layers of lunar dust. Although algorithms tend to perform well on training datasets, Crater Detection Algorithms (CDAs) often exhibit weak generalization when applied to new patches or other bodies of data. The complexity of the crater, extreme variations in form and lighting, the orders of magnitude size differences, overlap, and decline make it difficult to build trustworthy CDAs. A promising two-step CDA involving hypothesis generation and verification was proposed by Emami et al.^10^. Crater identification is difficult due to their huge size differences, extensive form alterations, and frequent occurrence. The crater's area is boxed with boosting and defined the crater's boundaries with a Hough transform by Robbins et al.^11^. Among the techniques used to find craters are the Hough transform, elliptical fitting, genetic algorithms, Gist features, watershed transform, pattern recognition, radial consistency algorithms, and combinations of these methods^12^. The Deep Moon model for crater detection on the lunar surface, which is based on the U-Net model of image semantic segmentation in deep learning was suggested by Silburt et al.^13^. The model was then used on the surface of Mercury to identify craters, and the findings were satisfactory. To facilitate the rapid identification of craters on the surface of Mars, the Deep Mars model was designed by extending the Deep Moon model structure to Martian craters by Lee^14^. CNN in deep learning in combination with a custom meteorite crater sample database to find large meteorite craters on the lunar surface was employed by Lei, et al.^15^. Template Matching-based algorithm using the edge detection method was proposed by Flores-Méndez^16^. Probability volume analysis obtained by template-matching-based algorithm was proposed by Banderia et al.^17^. Autonomous crater detection algorithm using multi-resolution feature point extraction and crater detection was proposed by Meng et al.^18^. A combinational approach that employs multiple methods to enhance the adaptability to various crater sizes was recommended by Sawabe et al.^19^.

The novelty of the model proposed by the authors in this study lies in the following points:

- Custom semantic segmentation based on the U-Net model with (Resnet18) is a novel approach to automate the detection of lunar craters, which has not been used before on the Mars-Lunar crater dataset.
- The model uses a pre-trained Resnet18 as a backbone on the ImageNet dataset, which allows for faster and more efficient training.
- The model leverages the best-performing pre-trained model, specifically trained on a vast and varied dataset, to achieve superior performance on the TMC-2 DEM images from the Chandrayaan-2 Mission. This ensures that the model has acquired comprehensive and broadly applicable characteristics to efficiently detect craters on the surface of the moon. The model gains from the previous knowledge and representations acquired from the ImageNet dataset, which includes a variety of object types, by utilizing the pre-trained model.
- By sharing full-image convolutional features, the detection network and the region proposal network increase object detection accuracy. The pre-trained model's incorporation into the novel semantic segmentation method—which is based on the U-Net model—further improves detection powers. The fusion of the pre-trained model's high-level features with the U-Net's ability to preserve detailed information facilitates accurate and comprehensive crater detection.
- Overall, this approach allows for a more detailed and accurate representation of the lunar surface than was previously possible and has important implications for future exploration and scientific research. This fusion results in a very efficient method for utilizing the TMC-2 DEM data from the Chandrayaan-2 Mission to find craters on the lunar surface.

Following is an outline of this paper's structure: A thorough literature assessment is provided in “Historical background of the development of crater detection algorithm” section, with an emphasis on earlier studies in deep convolution neural networks and the detection of craters on Mars and the moon. A thorough discussion of relevant research is provided. The difficulties or obstacles that the data set presents are covered in “Dataset” section. An overview of the dataset used in the current study is given in “Dataset” section. The methodology is presented in “Model description, Methods, techniques, studied material, and area description” section, which offers a thorough justification of the selected course of action. The experimental results are given in “Results” section and discussed in “Discussion” section. Limitations of our model are provided in “Limitations” section. “Conclusion” section offers concluding remarks, summarizing the findings and their implications while providing a comprehensive discussion of the research conclusions drawn and discusses the future scope of the research.

## Historical background of the development of crater detection algorithm

There have been several research studies and publications related to this field. Here are some examples of related work on lunar crater detection described. The use of a type of artificial intelligence called CNNs to detect lunar craters on the moon was explored by DeLatte, et al.^20^. A technique was presented to train a CNN to automatically detect craters in photographs of the surface of the moon by learning its characteristics, which include its round form and sharp edges. Previous crater-based terrain relative navigation (TRN) systems identified craters from visual imagery using conventional image processing methods. These methods, however, are frequently not resistant to alterations in camera settings, angles for viewing, and illumination. Recent studies have demonstrated that by using advancements in computer vision and deep learning, automated crater detection may significantly enhance robustness^21^. CNN was introduced in 2012, and as neural network-based image segmentation methods gained popularity, the relevant evaluation procedures to assess the impact of craters in the charge coupled device (CCD) photos of Chang by Jia et al.^22^. A methodology for lunar crater detection and classification using CNNs was proposed. Utilization of a dataset of high-resolution lunar images and training a CNN model to identify and classify craters based on their size and morphology. Specializing in crater recognition on the lunar surface using the support vector machine (SVM) method, high accuracy in recognizing and categorizing lunar craters is obtained, indicating the efficacy of CNNs for this task. The SVM classifier utilized the points of interest and relevance of each crater to create a significant feature vector. The SVM vector was then utilized to create impact craters and classification criteria that identified impaling objects^23^. They extracted various features from lunar images, such as color, texture, and shape descriptors then trained an SVM model on a labeled dataset to detect craters. The study demonstrates the feasibility of using SVM for lunar crater detection and achieves promising results^23^. The work presents a methodology based on traditional image processing techniques for lunar crater detection. Filters, segmentation, and morphological operations to enhance lunar images and identify crater-like structures were employed. The study evaluates the effectiveness of different image processing techniques for crater detection and discusses their limitations compared to more advanced machine learning approaches. The study examines many deep-learning architectures for crater identification on the moon. The subject of study was the effectiveness of many CNN models, including AlexNet, VGGNet, and ResNet, on a sizable collection of images^24^. By leveraging the spatial distribution and characteristics of the clusters, they achieve reliable crater detection without the need for labeled training data. These related works highlight the application of CNN and alternative methodologies for lunar crater detection. Each study explores different techniques, ranging from traditional image processing methods to machine learning approaches, demonstrating the diversity of approaches in this field^25^. The findings assist in the development of reliable and effective methods for detecting lunar craters, which can facilitate exploration and research of the lunar surface—a technique for locating craters in lunar mare regions and determining their ages^26^. The method uses image processing techniques and machine learning algorithms to locate craters and estimate their ages based on physical features. Using image processing techniques, the photographs' contrast is enhanced, and the crater-specific characteristics are brought to light. The features are then categorized, and the craters are located using machine learning methods. A deep learning strategy based on grid partition for crater detection on the lunar surface was suggested by Hashimoto and Mori^27^. The process creates a grid out of the lunar surface and employs a convolutional neural network to find craters in each grid cell. To teach the network the characteristics of craters, a sizable data collection of lunar photographs was used for training.

## Dataset

**Figure 1:**
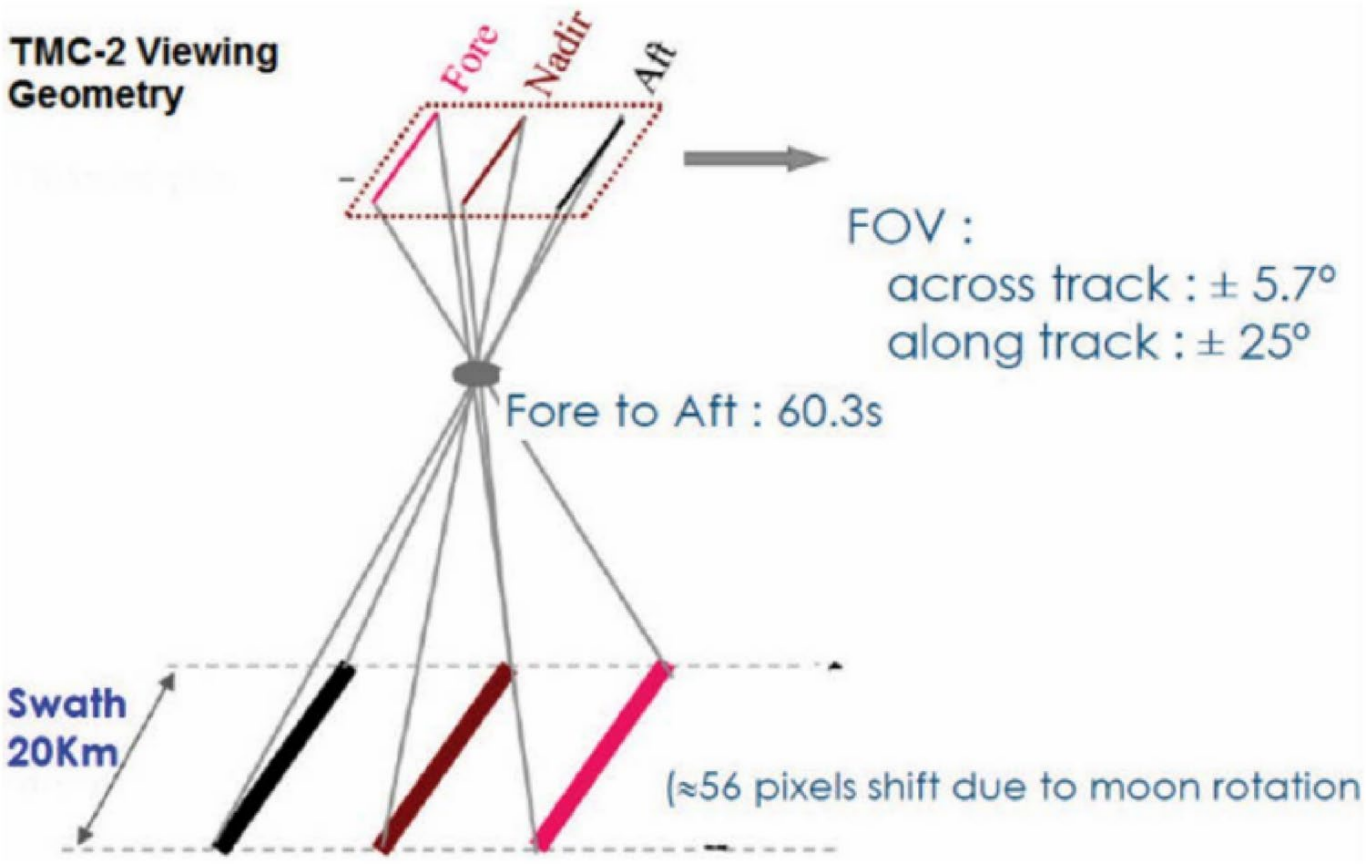
TMC-2 principle of operations^29^.

TMC-2 ortho and LRO NAC images (obtained from the RoboFlow) have been used during the training phase and testing phase of the proposed model while for validation of the model, only TMC-2 ortho images have been used. Technical details of TMC-2 and LRO NAC images are as follows:

TMC-2: The Terrain Mapping Camera (TMC) is a high-resolution camera that was created to map the terrain of the Moon's near and far sides. Images for TMC-2 are sourced from ISRO’s Chandrayaan-II (issdc.gov.in) Pradan Website^28^. TMC-2 is an instrument component onboard on Chandrayaan-II Mission. A thorough 3D atlas with a spatial and elevation resolution of 5 meters is being made as the main objective to better comprehend the lunar surface and the processes affecting it. TMC has a spatial resolution of 5 meters, a 20-km swath from 100 km lunar polar orbit, and functions throughout the 0.5–0.85 m panchromatic spectral range. Fore, nadir, and aft views of the satellite's ground track are recorded by the camera using a push-broom imaging mode and three linear 4k element detectors. The fore and aft view angles are 25 degrees apart from the nadir. TMC has a dynamic range that can capture both newly formed crust rocks and fully developed mare soil, and it detects the solar radiation that is reflected or scattered from the Moon's surface. Leveraging the proven performance of this model, it is further utilized for detecting lunar craters using TMC-2 (Terrain Mapping Camera-2) images. Figure 1 shows the schematic of the principle of operations of TMC-2. FOV (Field of View) is the angular view which camera observes at a given time.

LRO NAC: The lunar reconnaissance orbiter (LRO) is equipped with a unique set of three cameras known as the lunar reconnaissance orbiter camera (LROC). The LROC captures high-resolution black-and-white images, along with moderate-resolution multi-spectral photos of the lunar surface, providing an invaluable resource for lunar exploration and research. The LROC primarily consists of two narrow-angle cameras (NACs). These cameras are specifically planned to produce 0.5 meter-scale panchromatic images across a stretch of 5 km on the moon's surface, enabling detailed visualization of the lunar topography. In addition to the NACs, the LROC also features a wide-angle camera (WAC). This camera is designed to generate images at a scale of 100 m/pixel in seven distinct color bands, covering a broad swath of 60 km on the lunar surface. This provides a broader context and an overview of the lunar landscape. Lastly, the sequence and compressor system (SCS) works in tandem with both the Narrow Angle and Wide-Angle Cameras, aiding in efficient data collection. The compressor makes it possible to manage and process the vast amounts of data generated by the high-resolution cameras, ensuring seamless operation of the LROC system^30^.

As annotated TMC-2 images were unavailable, the approach was trained and tested on Mars and Lunar crater images obtained from the RoboFlow augmentation model. This dataset includes images and labels of the surfaces of Mars and the Moon, which may contain craters. The data source has a mixture of Mars images from ASU and USGS, and moon images are from NASA’s Lunar Reconnaissance Orbiter mission LROC NAC^31,32^. The public dataset of Mars-Lunar craters had been annotated for object detection purposes and sourced from Roboflow Universe^33^. The images have been pre-processed using RoboFlow to remove EXIF rotation and resize to 512 × 512. Each image comes with a labeling file in YOLOv5 text format that we created ourselves for object detection purposes. Additionally, we provide a trained YOLOv5 model file for each new version of the dataset using the latest version of the data. The current network structure used is YOLOv5m6. We have used 143 images of the Mars_Lunar dataset^33^ for training when performing a train-test-validation split and craters are annotated in YOLO v5 PyTorch format. The total number of craters in the training dataset is 1034. Mars_Lunar dataset is used to illustrate the efficiency of transfer learning in the performance of the model as well as make our model generic.

Lunar Dataset Details:

The model was trained using only the lunar dataset for the unannotated TMC 2 dataset. For which the details are provided below:

- Total images—3556
- Training set—2310 images i.e., 65%
- Testing set—890 images i.e., 25%
- Validation set—356 images i.e., 10%
- Total number of craters—7048
- Image size—416X416
- The Lunar Dataset collected to train the model proposed is from Roboflow^34^

Chandrayaan 2 Dataset Details:

The model was then trained-tested and validated using only the lunar dataset for the annotated TMC-2 dataset. For which the details are provided below:

- Total images—500 images augmented to 2000 images.
- Training set—1400 images i.e., 70%
- Testing set—400 images i.e., 20%
- Validation set—200 images i.e., 10%
- Subsets—24 each containing either 83 or 84 images

The model was tested using Chandrayaan 2 TMC 2 data from random locations of the moon. Images that were tested were of different locations.

**Figure 2:**
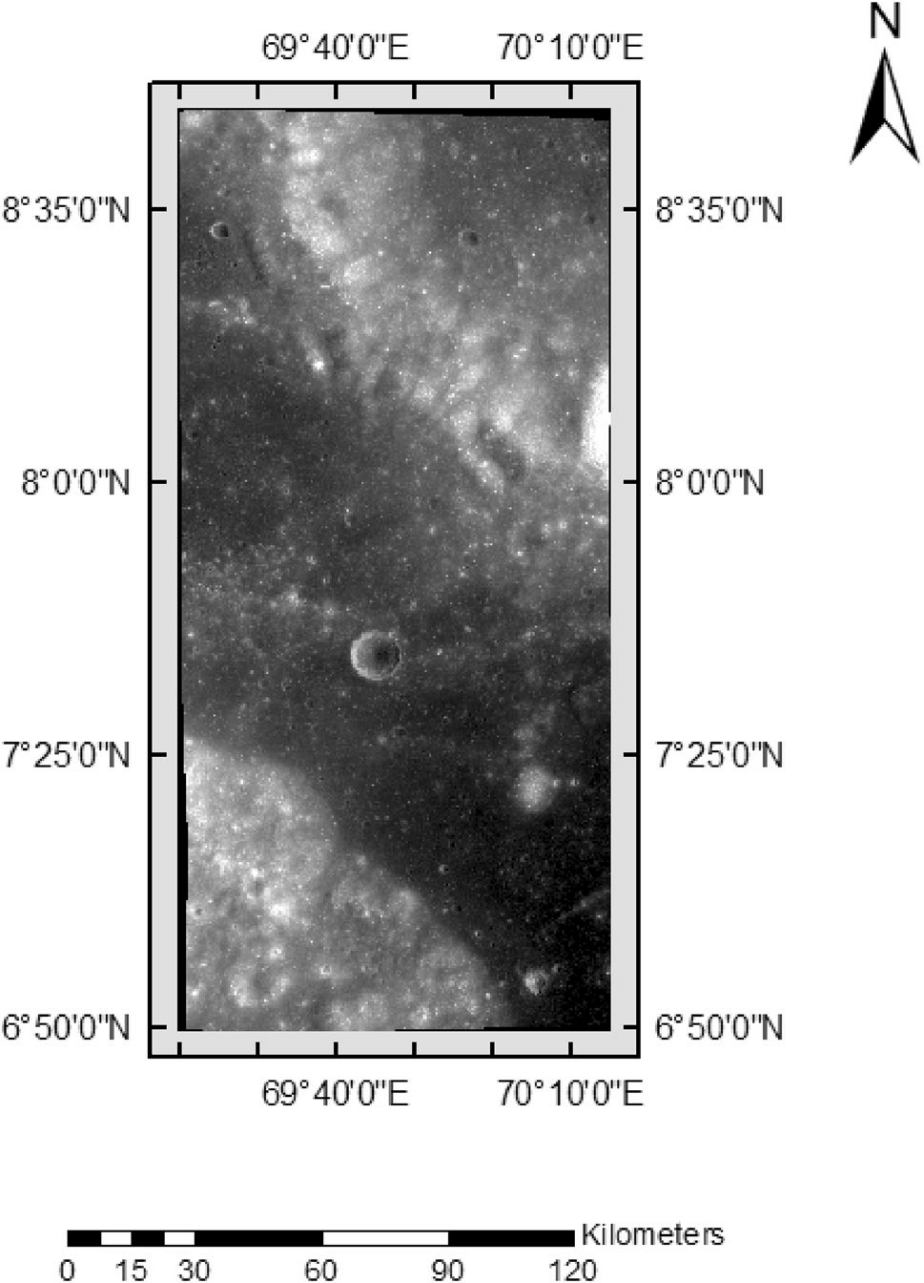
Image Sample having ID—ch2_tmc_ndn_20220803T0532523437.

**Figure 3:**
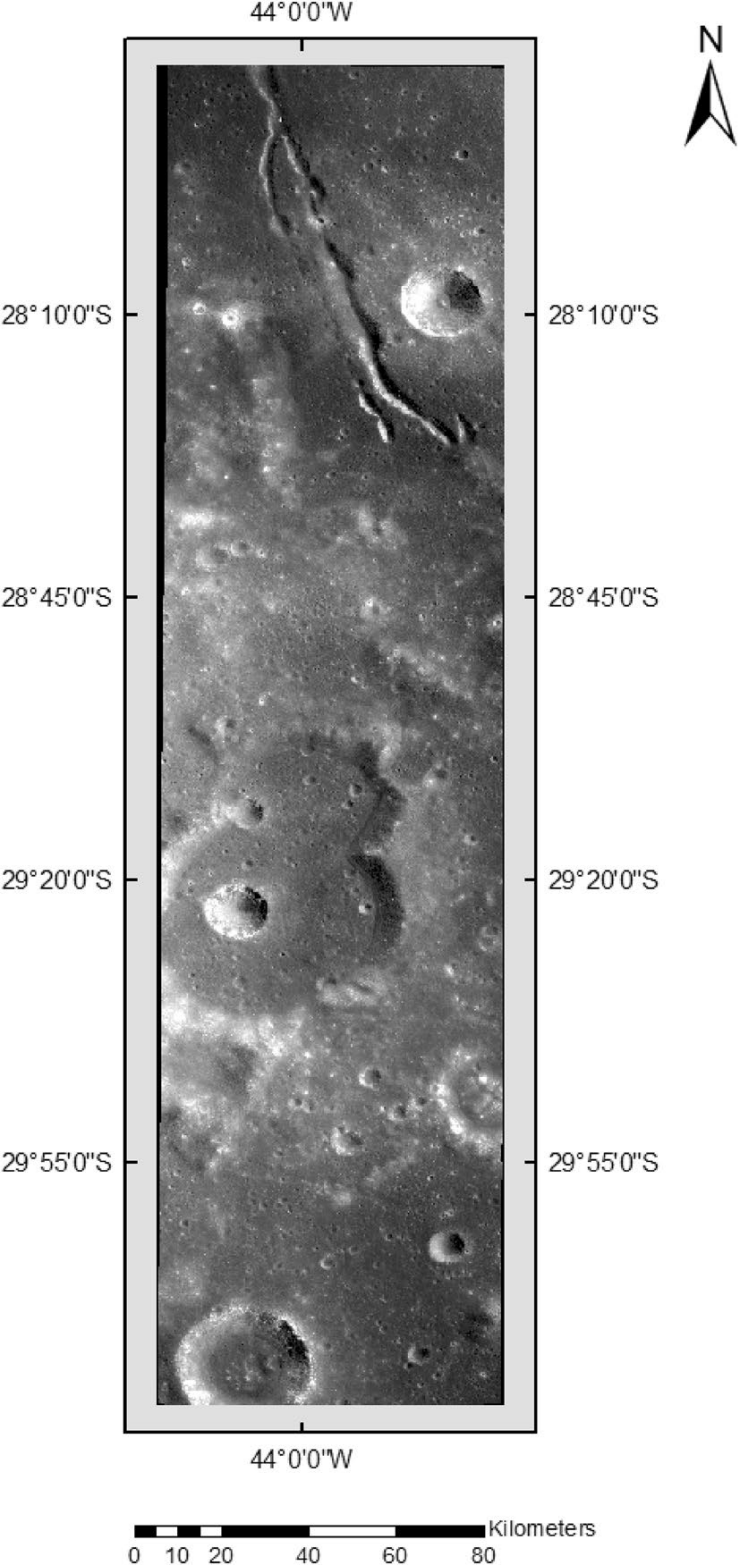
Image Sample having ID—ch2_tmc_ndn_20200209T0032589097.

Some samples used are given in Figs. 2 and 3 with their coordinates.

Data preprocessing is a very crucial step in the development of a deep learning model. It involved preparing image datasets for training, augmentation to increase the diversity of data, and implementing techniques like k-fold cross-validation to ensure robust evaluation of the model. To ensure uniformity and standardization, the images are resized to a particular resolution, such as 256 × 256 or 512 × 512 pixels.

Normalization is performed to scale the image pixel values to a common range, such as [0,1] or [− 1,1]. This phase reduces the impact of lighting and contrast differences, allowing the model to concentrate on learning structural features rather than pixel intensity variations. Data augmentation techniques, including rotation, scaling, inversion, and the addition of random noise, are used to generate additional training images and increase dataset diversity. We divided the annotated dataset into multiple subsets or folds using k-fold cross validation, to ensure that the model is trained on a substantial portion of the data and that distinct images are available for objective evaluation. To prevent performance bias, care is taken to sustain the distribution of crater and non-crater images in both sets.

Roboflow is used for augmenting the dataset which means augmentation is applied to existing images in the dataset to improve the ability of the model to generalize and perform well for new or unseen images. Augmentations are grouped and randomized with settings and values for each setting are applied to each augmented image. Duplicate images are also filtered in the process. Image augmentation is applied to only the training dataset^35^.

Roboflow helps in labeling/annotating the craters by using the bounding box. A bounding box, or the rectangle that surrounds an object in an image, defines each object. The width, height, and x and y coordinates of the top-left corner of the box, along with its coordinates, define the bounding box^36^.

- The discovered object’s class label (all objects in this instance have the same label, 0).
- The bounding box’s top-left corner’s x-coordinate, normalized by the image’s width
- The bounding box’s top-left corner’s y-coordinate, normalized by the image’s height
- The bounding box’s width, adjusted for the image’s width
- The bounding box height, scaled according to the image’s height

However, the Roboflow library is commonly used for data augmentation and pre-processing in computer vision tasks and may have been used in the training pipeline for the object detection model. Roboflow is a platform for managing, annotating, and augmenting image datasets. It provides various tools for data annotation, data pre-processing, and data augmentation. The platform supports various popular image formats and provides an easy-to-use interface for managing datasets. Roboflow also offers extensive integration with popular deep-learning frameworks like TensorFlow, PyTorch, and Keras. On the other hand, Albumentations is a Python library for image augmentation in machine-learning experiments. It provides various image transformation techniques like flipping, rotating, scaling, cropping, and color changes. It is designed to be fast, flexible, and easy to integrate with popular deep-learning frameworks like PyTorch and TensorFlow. It supports both CPU and GPU processing, and its augmentations are compatible with both NumPy and PyTorch tensors. Roboflow is more of a platform for managing and pre-processing image datasets, while the Albumentations library is specifically focused on image augmentation for machine learning.

Since our dataset comprises different quality images (Mars and Lunar), the model is trained for various image qualities. Due to this our model can be tested by any quality of image.

**Figure 4:**
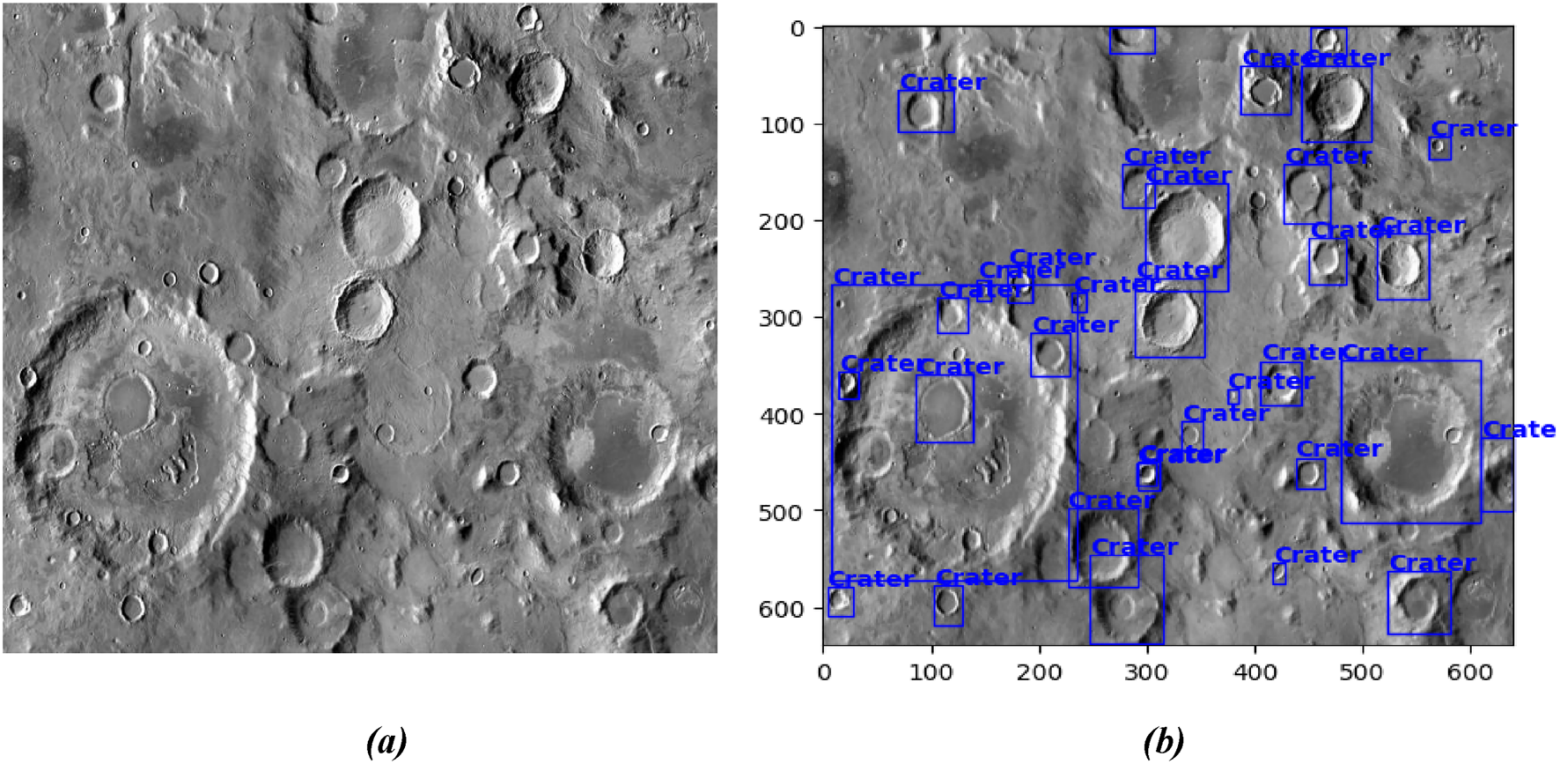
(**a**) Presents the original image of the Lunar surface and (**b**) describes annotations.

Figure 4a is the original image of the Moon surface from the Mars_Lunar Dataset. Figure 1b presents the labeled feature using Roboflow, the X and Y-axis are the pixel values of the image where each pixel is 0.5–2 m.

A dataset of 2000 TMC 2 images has been annotated with crater locations and sizes by human experts and is currently used for training and testing crater detection algorithms. The dataset covers various regions of the Moon, including the nearside and the farside, and different types of terrain, such as mare, highlands, and polar areas. The dataset is divided into 24 subsets, each contains 83 or 84 images, for cross-validation. Cross-validation is a technique that splits the dataset into multiple subsets and evaluates the performance of the algorithm on each subset, using the rest of the data for training. This helps to reduce the bias and variance of the evaluation and to estimate the generalization ability of the algorithm.

**Figure 5:**
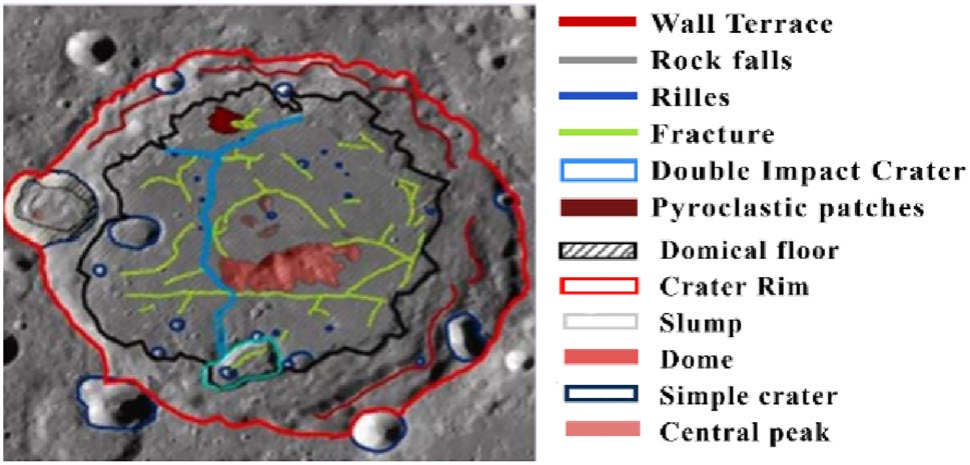
Morphological parameters analysis.

Different types of difficulties present in the image for accurately identifying their features:

1. Overlapping, nested, and small-shaped lunar craters present difficulties in accurately identifying their features. Their description is given in Table 1. Ensuring consistency is challenging for quantifying the data into algorithms.
2. Identifying and measuring parameters defining crater shape accurately is complex. Challenges arise in distinguishing craters from other formations, determining various parameters, and addressing limitations in lighting conditions and data quality. Morphological characteristics on the lunar surface presenting challenges in the identification of craters are analyzed in Fig. 5.
3. Lack of Ground Truth: The absence of annotated data means that there is no ground truth for model training and evaluation. The models must rely on inherent patterns in the data to learn and generalize lunar surface features.
4. Visualizing craters across diverse datasets is complicated by variations in lighting and shadow rendering regions which is shown in Table 2. Accurately defining crater boundaries and coverage becomes complex due to overlaps and dataset variations, impacting comparability. To overcome this challenge during the learning phase in the augmentation task saturation was adjusted between − 50 and + 50%, brightness was adjusted between − 40 and + 40%, and exposure was adjusted between − 50 and + 50%. This helped to identify craters visible in different regions.

**Table 1:** Types of craters causing difficulties.

S. no	Type of crater	Description	Sample image
1	Overlapping crater	A crater on which other craters are partially or overlapped on it	
2	Nested crater	A primary crater formed and smaller secondary craters from inside it	
3	Small craters	Circular depressions on the surface of celestial bodies are formed by the impact of small meteoroids or asteroids. Usually less than 15 km (9 miles) in diameter and have a simple bowl-shaped structure. They are more abundant than large craters

**Table 2:**
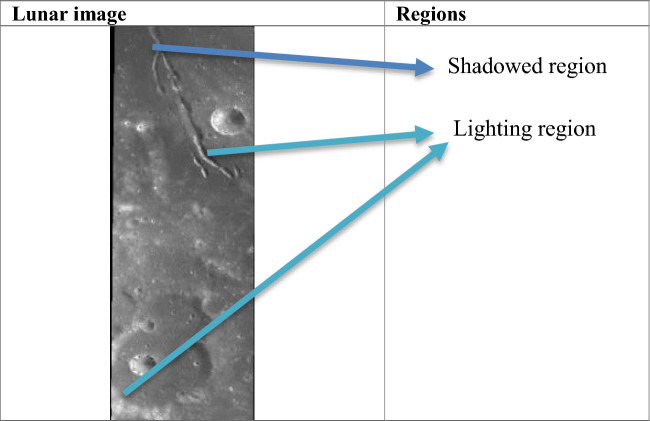
Different visualization regions cause difficulties.

## Model description, methods, techniques, studied material, and area description

### Proposed model description

**Figure 6:**
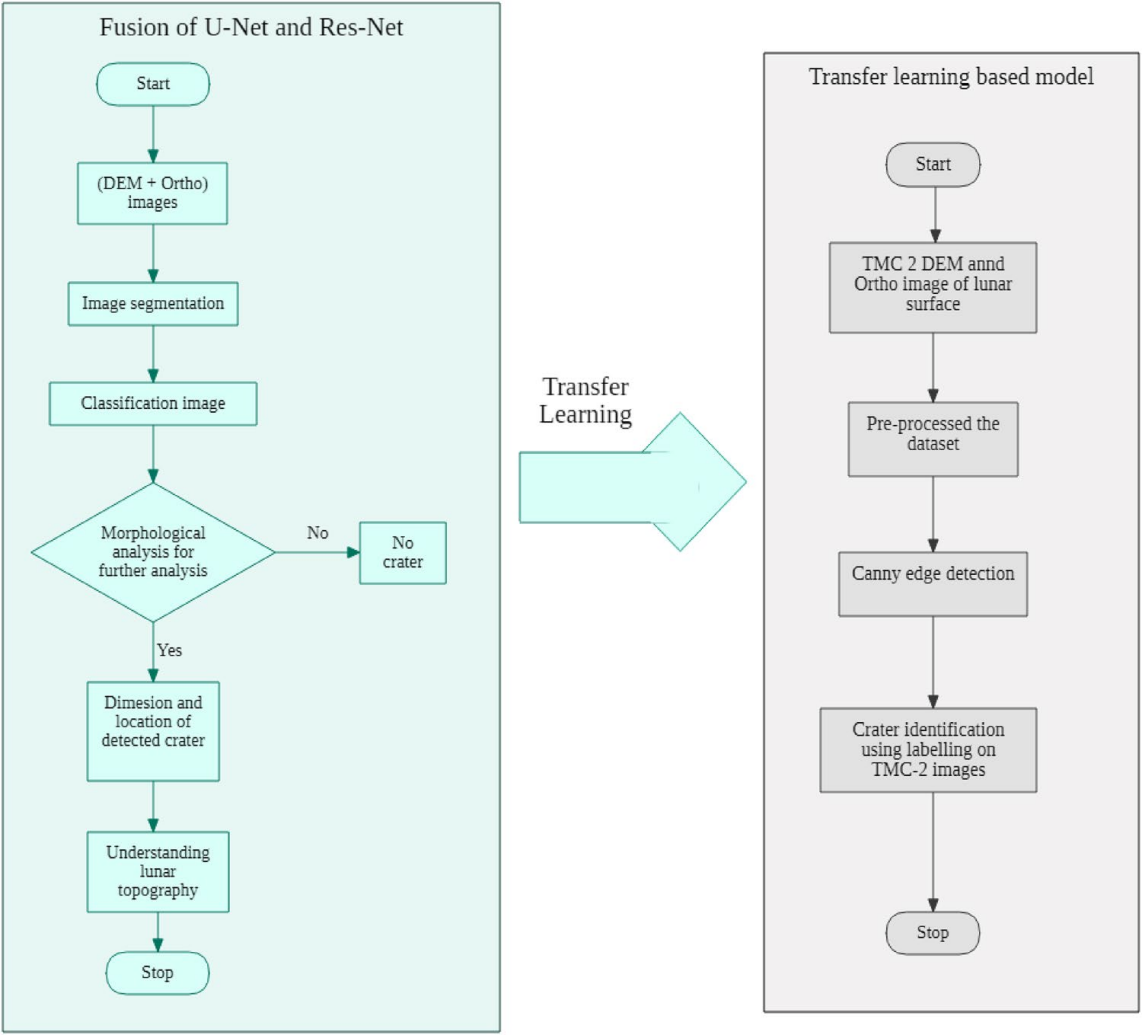
Flowchart of the proposed model.

The deep learning model was divided into two parts: initially, it was trained using the Resnet18 technique as the backbone and U-Net based on Lunar Images obtained from LRO NASA’s mission^34^ for obtaining a Pre-trained Model. With the help of Transfer Learning, the pre-trained model and the Canny Edge Detection Algorithm bounding boxes were used to identify circular objective craters in the images from TMC-2^37^. Figure 6 depicts the overall flowchart of the proposed model.

We have trained our model on NASA lunar images which are labeled and annotated and fine-tuned it to detect craters in lunar images taken by ISRO, which are unlabelled.

- We used Transfer Learning on a pre-trained model to identify craters in the images from TMC-2^37^.
- After all the pre-processing steps including Canny Edge Detection and Hough transform, the U-Net model predicts a mask where each pixel is labeled as “crater” or “non-crater”. This is usually done for crater spots ranging between 10 and 40 px with the help of this we can obtain craters starting range is 10 m.
- The location and dimension of each of the craters found in the image using post-processing of the image, are then converted into geographical coordinates, thus the repetitive craters after resolution in different images can reappear which are separated based on geographical image coordinates on the pixel map.

### Methodology

**Figure 7:**
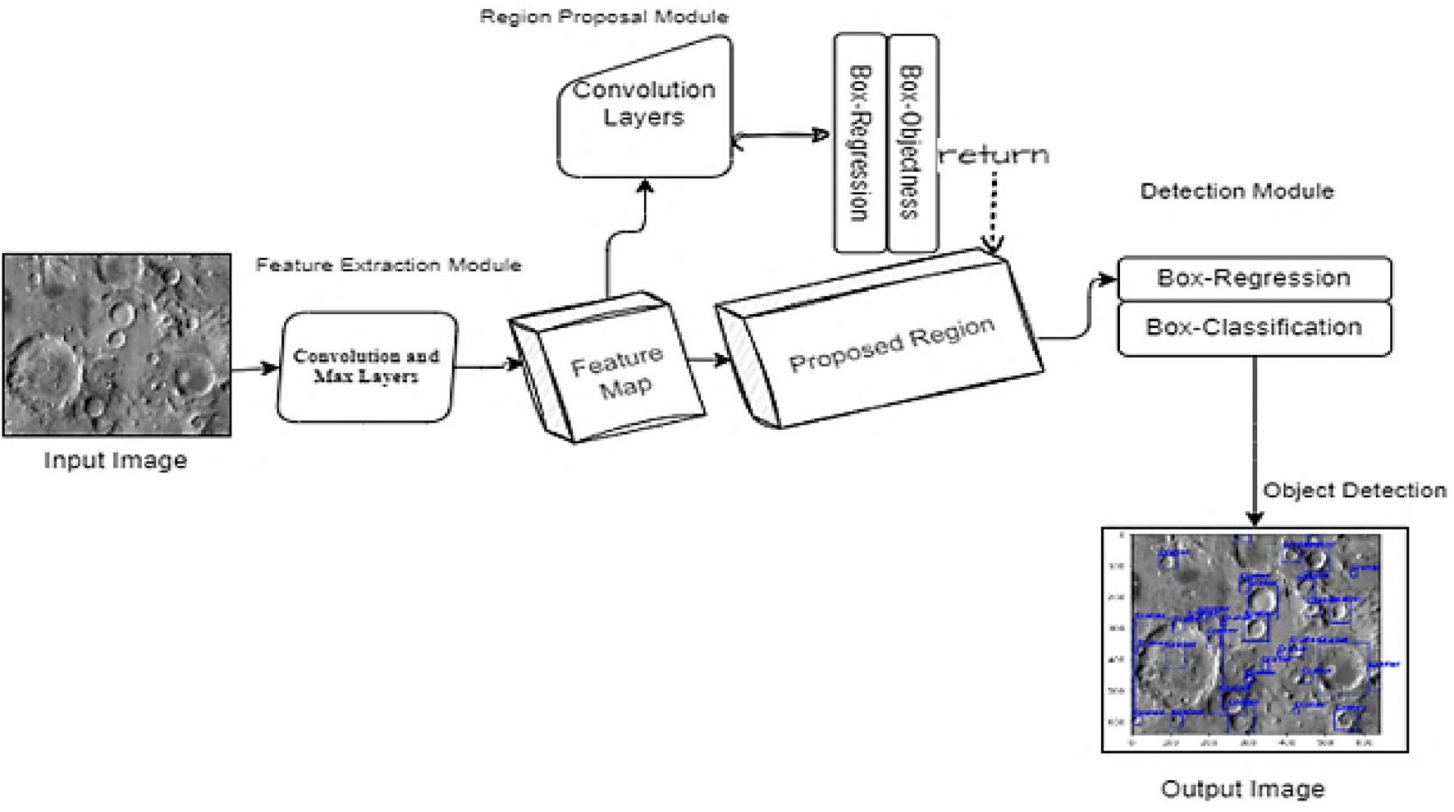
Model architecture of the designed approach for crater detection.

*Deep Learning* The primary objective of deep learning, a branch of machine learning, is to train multi-layered artificial neural networks to understand and predict complicated data. Speech recognition, natural language processing, and computer vision are just a few of the fields it has transformed. By automatically deriving hierarchical representations from data, deep learning models may extract significant characteristics and generate precise predictions. Deep learning models acquire the ability to automatically extract features and patterns from the input data during the training phase. The technique known as backpropagation is used to do this, and it entails modifying the neural network's weights and biases to reduce the discrepancy between the true and expected outputs. The model can identify the ideal set of parameters through this optimization process, which best captures the underlying patterns in the data^26^. Computer vision tasks including segmentation, object identification, and picture classification have been transformed by deep learning. Image identification challenges have shown amazing accuracy for convolutional neural networks (CNNs), a sort of deep learning architecture particularly built for evaluating visual input. Deep learning does, however, present several difficulties. Both a large amount of labeled data and a significant amount of processing power are needed for deep neural network training. Deep learning algorithms are prone to overfitting due to their complexity, which occurs when the model performs well on training data but not on fresh, unknown data. Regularization techniques and larger datasets are often employed to mitigate overfitting. Overall, deep learning has had a transformative impact on various fields, enabling breakthroughs in image analysis, natural language understanding, and many other areas. It continues to advance the frontiers of AI and holds promise for solving complex problems and driving innovation in the future. Figure 7 illustrates the model architecture designed specifically for crater detection here in the figure using Mars_Lunar dataset images. The U-Net architecture is used, which has an encoder route that records high-level characteristics while progressively decreasing the spatial resolution. The decoder route, on the other hand, oversees upsampling the features to return the original spatial resolution. By adding skip links between the respective levels of the encoder and decoder, it is possible to combine high-level and low-level data, which guarantees precise and thorough crater identification in Mars_Lunar dataset images.

**Figure 8:**
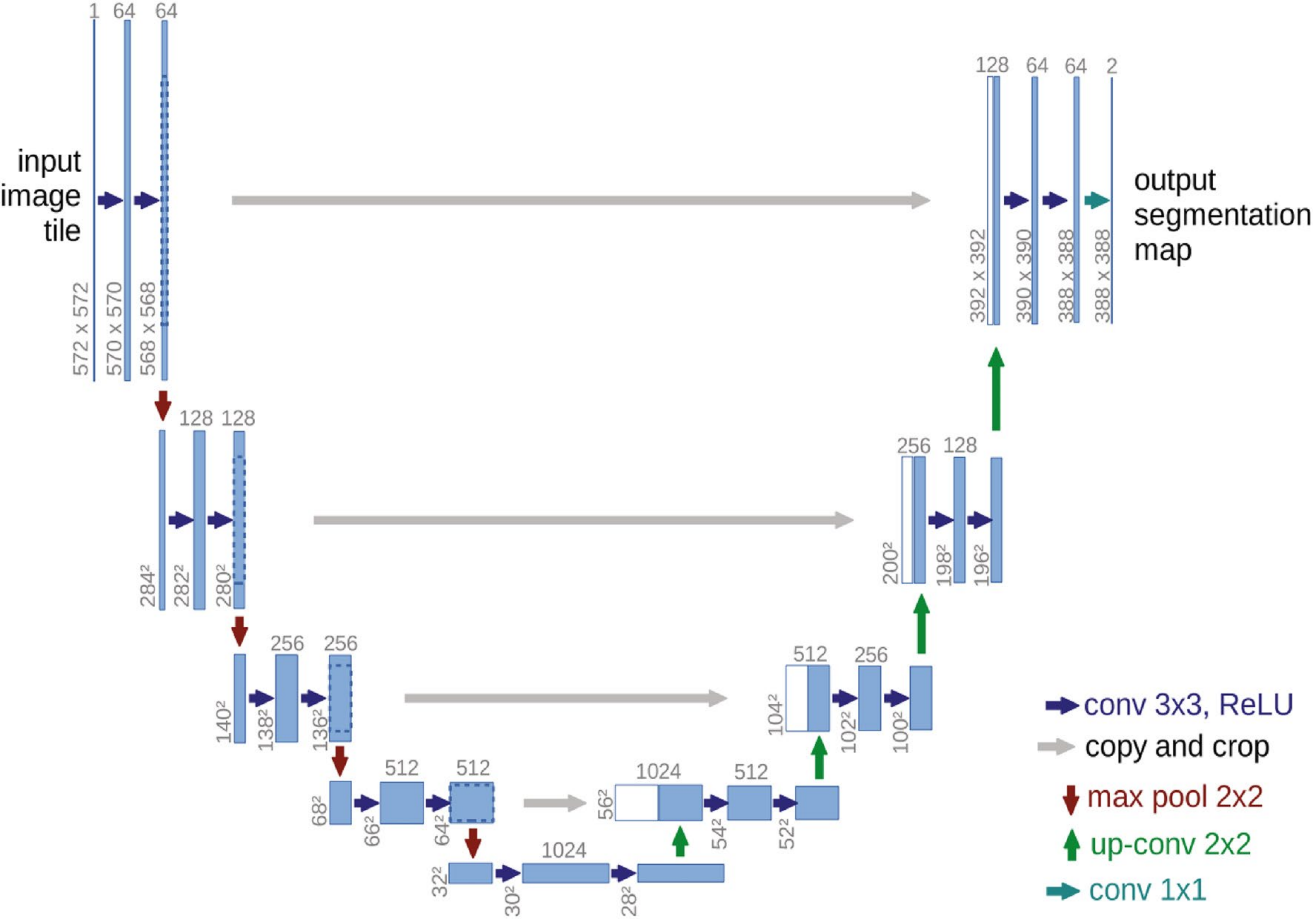
Modified U-Net architecture developed by^39^.

*U-Net* A common architecture for image segmentation tasks is U-Net. It comprises a decoder pathway that upsamples the features to regain the spatial resolution and an encoder pathway that progressively diminishes the spatial resolution while collecting high-level information^38^. U-Net joins equivalent levels of the encoder and decoder via skip links, allowing low-level and high-level information to be combined. It has been extensively utilized for applications like object identification and biological picture segmentation. Overall, U-Net's architecture, with its skip connections, symmetric design, efficient parameter usage, and versatility, has established itself as a powerful tool for image segmentation tasks, providing accurate and detailed segmentation results in various domains. The authors use this model for training the Mars and Lunar images. Modified U-Net architecture^39^ developed by (Olaf Ronneberger et al.) has been used in our proposed model. Figure 8 illustrates the model developed by Olaf Ronneberger.

*FCNN (Fully Convolutional Neural Network)* FCNN is a type of neural network architecture designed for pixel-level prediction tasks such as semantic segmentation. Unlike traditional convolutional neural networks (CNNs) that output a single label for the entire input image, FCNNs produce dense predictions by preserving the spatial resolution of the input. FCNNs typically consist of convolutional and pooling layers for feature extraction and up-sampling layers for recovering spatial resolution. They have been successfully applied in various computer vision tasks, including scene parsing and object localization.

*Canny Edge Detection* Canny edge detection is a classic computer vision technique used for detecting edges in images. It was introduced by John Canny in 1986 and remains widely used due to its effectiveness. The Canny edge detection algorithm involves multiple steps, including noise reduction, gradient calculation, non-maximum suppression, and thresholding. It aims to identify significant changes in intensity, which correspond to edges in the image. Canny edge detection is useful for tasks such as image segmentation, object recognition, and feature extraction.

### Hybrid ResNet UNet model for crater detection in CH2 TMC2 images

To fulfill our objective we divide our model into two parts:

1. For training we utilize U-Net architecture based on resnet18 as the backbone of annotated images and labels obtained from the Roboflow augmentation library used especially for image augmentation purposes,
2. To detect craters, we have deployed a model, to achieve efficient detection of lunar craters on images acquired by TMC-2 on board the Chandrayaan-2 Mission.

**Figure 9:**
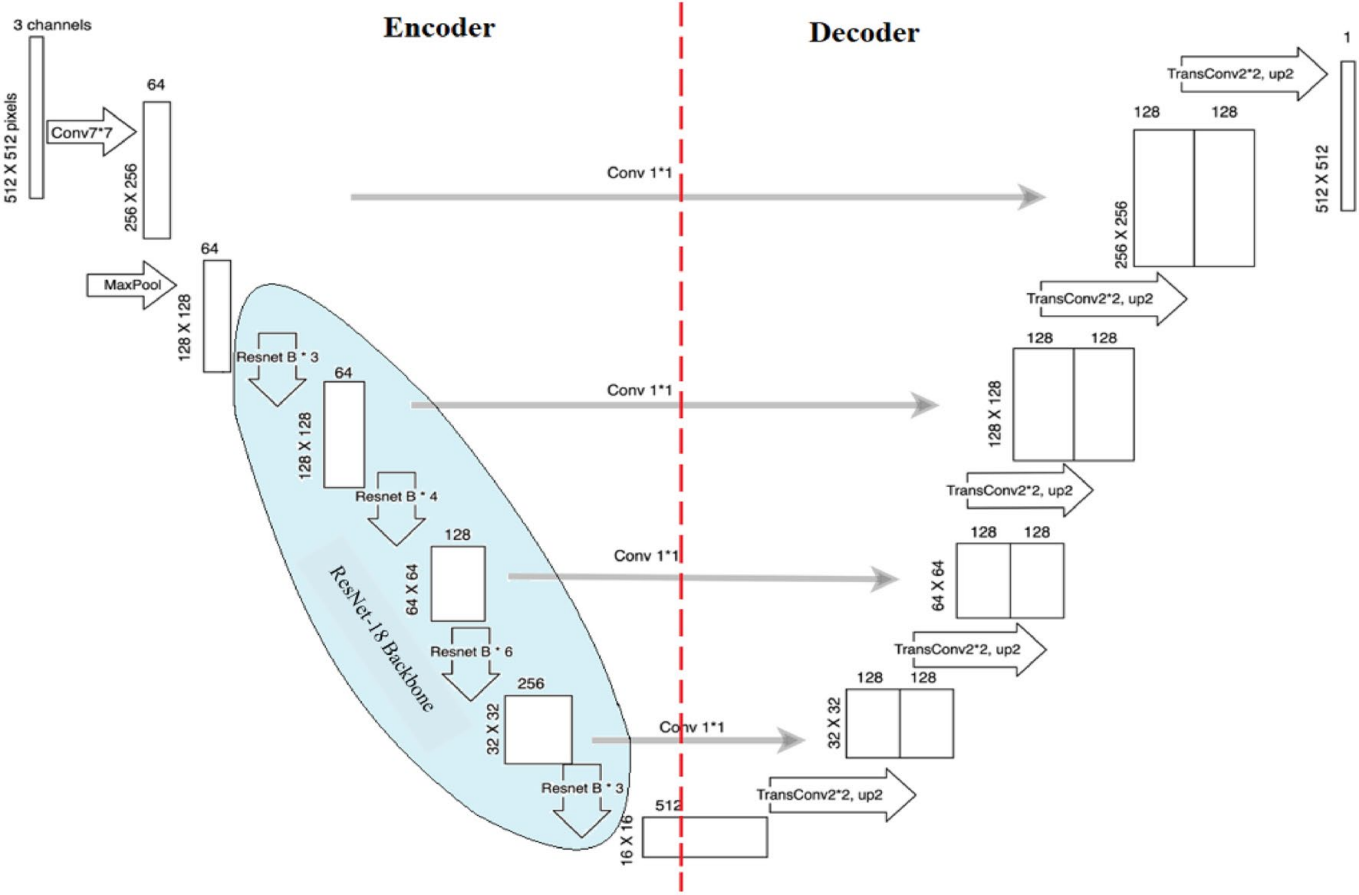
The architecture of UNet and ResNet-18 backbone as the proposed model.

In our proposed work, we implement a modified version of the UNET architecture, as shown in Fig. 9. The architecture comprises two main parts: an encoder on the left that reduces the spatial dimensions of the input, and a decoder on the right that restores the output to the original size. The two parts are connected by multi-level skip connections that transfer features across different scales. We use the Mars_Lunar crater dataset, which contains 143 images and corresponding labels in YOLO v5 PyTorch format, exported from Roboflow^33^.

In the proposed model custom implementation of the semantic segmentation U-Net architecture is performed. Using a symmetric expanding path to permit exact localization and a contracting path to collect context, U-Net is a popular deep-learning architecture for image segmentation problems. ResNet18, a well-liked deep convolutional neural network design with better accuracy while training on huge datasets than earlier models, serves as the foundation for this application. The model is optimized for the particular purpose of semantic segmentation using a cross-entropy loss function, having been pre-trained on ImageNet. Our model is trained using Customize semantic segmentation based on U-Net with ResNet18 as the backbone and ImageNet used as weights, for binary segmentation of images with 6 input channels and 2 output classes. The model is classified into two backgrounds and a crater. This specifies that weights pre-trained on ImageNet will be employed to initialize the pre-trained weights of the backbone network used in the U-Net design. This is useful because Image Net is a large-scale dataset with a diverse set of image categories, and pre-training on it can help the model learn better feature representations. We trained and tested our model on Google Colab Pro+ utilizing a GPU unit to achieve better computational performance. In this case, we used Python 3.7.4 version for the detection part, which corresponds to the 2nd part of our model. We can benefit from the characteristics that the model learned during the pre-training phase and refine them for the item identification task at hand by employing a pre-trained ResNet-50 backbone. The term “K-folds = 5” describes a kind of cross-validation method in which the data is divided into five “folds,” or equal-sized subgroups. The procedure is then done five times, using each fold as a test set once, with the model being trained on four of the folds and tested on the remaining fold. This can increase the model's overall resilience and aid in assessing how well it performs on various data subsets. The following formula may be used to normalize the image's shape (width, height) and coordinates (x, y):

1 $${\text{Normalized}}\_{\text{x}} = \left( {{\text{x}}/{\text{width}}} \right)$$

2 $${\text{Normalized}}\_{\text{y}} = \left( {{\text{y}}/{\text{height}}} \right)$$

The resulting normalized coordinates (Normalized_x, Normalized_y) will be in the range of [0, 1], representing the relative position of the point within the image.

A rectified linear activation unit (ReLU), a function that maximizes the product of its input vale and 0, follows a bank of filters that apply 3 × 3 padded convolutions to each convolutional layer of the design. In this work, we present a binary segmentation method for craters on planetary surfaces using a U-Net with a ResNet18 backbone. The 6-channel input, which consists of a collection of equivalent 3-channel feature maps and 3-channel pictures, is fed into the model architecture, which has been pre-trained on Image Net. There is no ignore index specified, and cross-entropy is the loss function that is applied. To facilitate training and evaluation of the model, we define a custom semantic segmentation task that inherits from the Semantic Segmentation Task class. This task includes a plot method for visualizing a sample from the dataset, which consists of an image, its corresponding labels, and the predicted segmentation output. During the training phase, the training step method performs one training step of the model using a batch of training data. The input image and its associated labels are taken out of the batch, the model's predicted output is computed, the cross-entropy loss function is used to compute the loss between the expected output and the ground truth labels, and the self.log technique is used to log the accuracy and loss. If the batch index is less than 10, the prediction is added to the batch, and the plot method is used to visualize the image, the labels that go with it, and the anticipated segmentation output. The test step method is used during the testing phase and calculates the loss and accuracy of the predicted output on a batch of testing data. The results are then logged using the self.log method. In this work, we investigate how transfer learning can be used to find lunar craters. We use the potency of pre-trained CNN models, like ResNet and ImageNet, which were initially trained using datasets from Mars and lunar images. We intend to illustrate the efficiency of transfer learning in enhancing the performance of crater detection models, especially when faced with little training data, by optimizing these models using lunar crater images. Our results demonstrate the noteworthy improvements made possible by transfer learning approaches in the context of the detection of lunar craters.

The algorithm for the program is given as follows:

- Step 1: Import required libraries.
- Step 2: Define constants and parameters.
- Step 3: Define transformations and utility functions including augmentation.
- Step 4: Mount the drive and extract the dataset.
- Step 5: Define a Custom Semantic Segmentation Task with training, validation, and testing steps.
- Step 6: Set up checkpoints, early stopping, and tensor-board logging.
- Step 7: Load the dataset and visualize a few samples.
- Step 8: Perform k-fold cross-validation.
- Step 9: Split the dataset into training and validation subsets
- Step 10: Create data loaders.
- Step 11: Create and initialize the U-Net model.
- Step 12: Define optimizer and learning rate scheduler.
- Step 13: Train the model for multiple epochs.
- Step 14: Evaluate the model on the validation set.
- Step 15: Set up testing parameters.
- Step 16: Load and evaluate the best model on the test dataset.
- Step 17: Perform inference on test images.
- Step 18: Load the model to evaluate.
- Step 19: Iterate over test images.
- Step 20: Perform inference.
- Step 21: Visualize and save the results.
- Step 22: Calculate and display the average FPS.

## Results

The absence of annotated data poses a unique set of challenges, as the models must autonomously identify and categorize lunar features without the guidance of predefined labels. Our evaluation metrics include average recall, precision, F1 measure, and accuracy, comparing the results obtained from models trained on data without annotations to those trained on annotated datasets. The challenges associated with the proposed approach contribute to this performance gap, highlighting the importance of annotated data for enhancing model accuracy and reliability in lunar surface analysis.

### Evaluation metrics

Details of evaluation parameters and metrics are shown in Tables 3, 4, 7 and 8 where ‘Area’ is the variable used in code that specifies the size of the craters to be detected with following details:

- all: This means that the code will detect craters of any size, regardless of how big or small they are.
- medium: This means that the code will detect craters that are between 5 and 15 pixels in diameter, which corresponds to craters between 1.25 and 3.75 km in real life.
- small: This means that the code will detect craters that are less than 5 pixels in diameter, which corresponds to craters smaller than 1.25 km in real life.
- large: This means that the code will detect craters that are larger than 15 pixels in diameter, which corresponds to craters larger than 3.75 km in real life.

**Table 3:** Performances of the model in terms of average recall (AR) for lunar crater detection (without annotation).

Subset no	IOU threshold	Area	Max detections	Average recall
1	0.50:0.95	All	1	0.45
2	0.5	All	10	0.63
3	0.75	All	100	0.85
4	0.50:0.95	Small	100	0.77
5	0.50:0.95	Large	100	0.79
6	0.50:0.95	All	100	0.87
7	0.50:0.95	All	1	0.60
8	0.5	All	10	0.66
9	0.75	All	100	0.76
10	0.50:0.95	Small	100	0.69
11	0.50:0.95	Large	100	0.80
12	0.50:0.95	Medium	100	0.79
13	0.50:0.95	All	1	0.57
14	0.5	All	10	0.70
15	0.75	All	100	0.60
16	0.50:0.95	Small	100	0.60
17	0.50:0.95	Large	100	0.62
18	0.50:0.95	Medium	100	0.89
19	0.50:0.95	All	10	0.43
20	0.5	All	100	0.52
21	0.75	Small	100	0.57
22	0.50:0.95	Large	100	0.67
23	0.50:0.95	Medium	100	0.70
24	0.50:0.95	All	1	0.56

**Table 4:** Performance of the model in terms of average precision (AP) for lunar crater detection (without annotation).

Subset no	IOU threshold	Area	Max detections	Average precision
1	0.50:0.95	All	1	0.57
2	0.5	All	10	0.61
3	0.75	All	100	0.59
4	0.50:0.95	Small	100	0.65
5	0.50:0.95	Large	100	0.68
6	0.50:0.95	All	100	0.67
7	0.50:0.95	All	1	0.50
8	0.5	All	10	0.56
9	0.75	All	100	0.65
10	0.50:0.95	Small	100	0.65
11	0.50:0.95	Large	100	0.64
12	0.50:0.95	Medium	100	0.67
13	0.50:0.95	All	1	0.60
14	0.5	All	10	0.57
15	0.75	All	100	0.58
16	0.50:0.95	Small	100	0.48
17	0.50:0.95	Large	100	0.57
18	0.50:0.95	Medium	100	0.60
19	0.50:0.95	All	10	0.60
20	0.5	All	100	0.50
21	0.75	Small	100	0.50
22	0.50:0.95	Large	100	0.58
23	0.50:0.95	Medium	100	0.56
24	0.50:0.95	All	1	0.50

The Max detections parameter is used to limit the number of bounding boxes that the model predicts for each image. It is set to 100 by default. This means that the model will output at most probable 100 bounding boxes for each image, regardless of how many craters are present in the image. The max detection parameter affects the performance of the crater detection algorithm in two ways:

- If the max detections are too low, the model might miss some craters that are present in the image, resulting in lower recall and precision scores. Recall is the fraction of true craters that are detected by the model, and precision is the fraction of detected craters that are true craters.
- If the max detections are too high, the model might output too many bounding boxes that are not craters, resulting in lower precision and higher inference time.

Inference time is the time it takes for the model to process an image and output the bounding boxes. Therefore, the optimal value of the max detection parameter depends on the distribution and density of craters in the images, as well as the trade-off between accuracy and speed.

The different values of IOU (Intersection over Union) are used to measure the accuracy of the object detection model. IOU is the ratio of the area of overlap between the predicted bounding box and the ground truth bounding box to the area of union between them. A higher IOU means a better match.

COCO metrics are evaluation criteria for object detection models. They were first proposed in the Microsoft COCO challenge by Lin et al. and have since been the standard evaluation criteria for object detection models. The code uses the COCO evaluation metric, which computes the average precision (AP) for different IOU thresholds. The rows of the result table show the AP for different IOU values, such as:

- 0.50:0.95: This is the mean AP over IOU thresholds from 0.50 to 0.95 with a step size of 0.05. This is the most comprehensive and robust metric, as it evaluates the model across a wide range of IOU values.
- 0.50: This is the AP for an IOU threshold of 0.50, which is equivalent to the PASCAL VOC metric. This is a less strict metric, as it only requires a 50% overlap between the predicted and ground truth bounding boxes.
- 0.75: This is the AP for the IOU threshold of 0.75, which is a stricter metric, as it requires a 75% overlap between the predicted and ground truth bounding boxes. This evaluates the model’s ability to produce precise localization.

### Analysis of data without annotation

Preliminary results indicate that the models trained on data without annotations exhibit lower average recall, precision, F1 measure, and accuracy compared to their counterparts trained on annotated datasets. Our findings underscore the significance of annotated data in training robust models for lunar surface analysis. While approaches show promise, further research is needed to address the unique challenges posed by the absence of ground truth labels.

Applying k-fold cross-validation is particularly beneficial when working with limited data, as it helps ensure that the model's performance evaluation is more representative and less dependent on a specific random split of the data. It also provides insights into the model's stability and generalization across different subsets of the data.

Table 3 presents the average recall of subsets, showcasing the model’s performance. Table 4 then illustrates the average precision of subsets in the proposed lunar crater detection model. Meanwhile, Table 5 provides insights into the model’s performance through F1 score and accuracy, complementing the already obtained average recall and precision values for subsets. The analysis reveals an accuracy of 80.96 and an F1 score of 0.7519.

**Table 5:** Performance of model based on F1-score and accuracy.

Subset no	F1 score	Accuracy(%)
1	0.5031	68.85
2	0.6217	73.81
3	0.6933	70.91
4	0.7032	78.65
5	0.7321	80.64
6	0.7519	80.96
7	0.5438	60.23
8	0.6196	68.12
9	0.7007	80.01
10	0.6569	78.53
11	0.7109	77.56
12	0.7208	80.47
13	0.6324	72.36
14	0.6257	68.61
15	0.5845	68.85
16	0.5228	57.84
17	0.5920	68.85
18	0.7203	75.77
19	0.5029	65.10
20	0.5111	60.26
21	0.5371	60.62
22	0.6203	70.06
23	0.6238	72.36
24	0.5276	60.26

**Figure 10:**
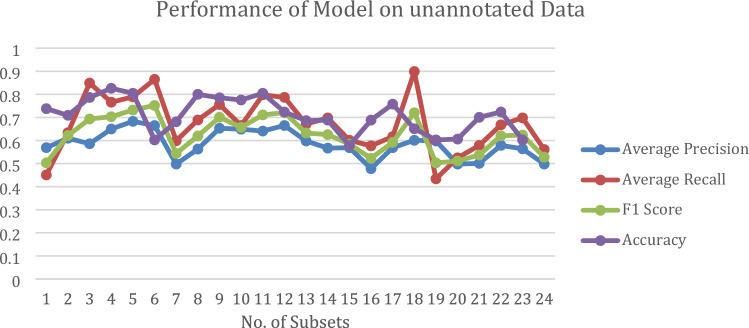
The Line graph displays the performance of the proposed model using unannotated data.

Figure 10 is a line graph depicting the performance of the proposed model using unrelated data. This graphical representation includes average precision, recall, F1 score, and accuracy across all epochs of subsets, with values ranging from zero to one.

In Table 6, the images originate from the TMC 2 sensor of Chandrayaan 2. The coordinates corresponding to these images are provided in Figs. 1 and 2. Notably, these images are unannotated and serve as new input for the proposed model to identify lunar craters. The ground truth craters are indicated with red circles, and it's essential to note that the X and Y axes denote pixel values within the image, not the actual geographical coordinates.

**Table 6:**
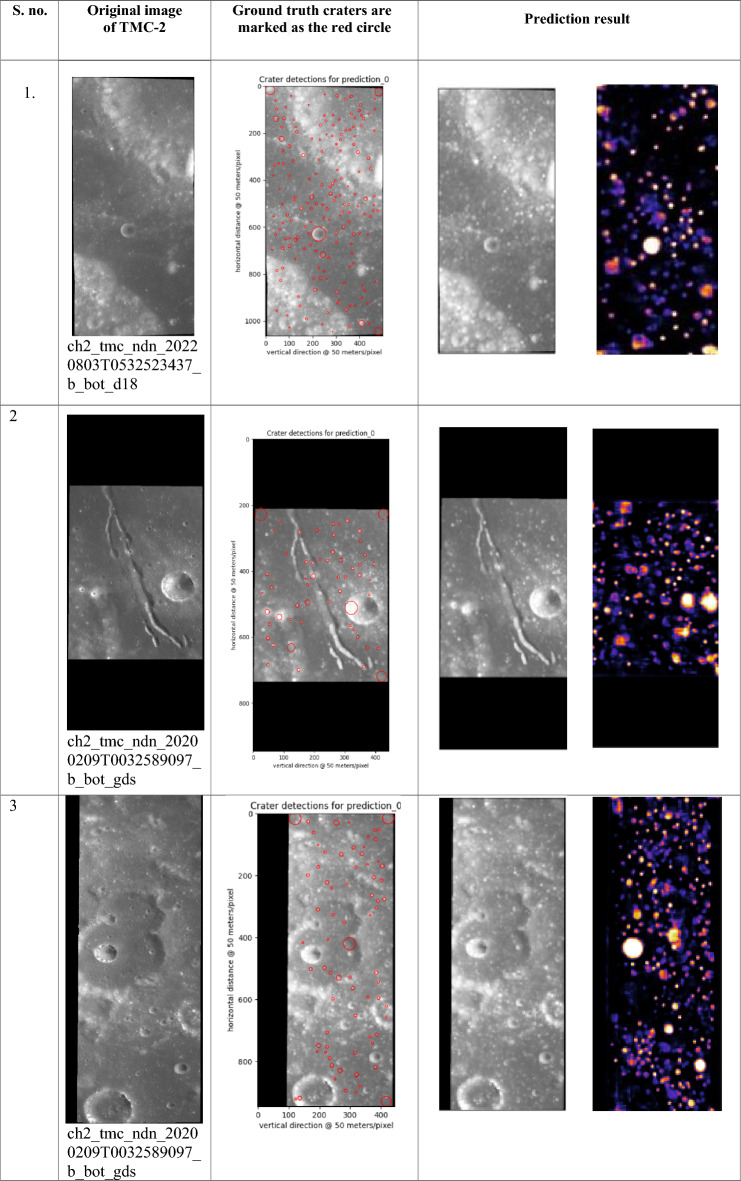
Pictorial representation of the detection phase without annotation.

In the “Prediction Result” column, the left image represents the original input, while the right image displays a heat map illustrating the model's predictions for crater detections.

### Analysis of data without annotation

However, the manual annotation of lunar surface data, a crucial step in training models, is a labor-intensive process, often constrained by time and resources. As a result, the availability of annotated data remains limited, presenting a challenge in achieving optimal model performance. Our focus lies on improving the performance of models designed for lunar surface analysis, where the acquisition of annotated data is a bottleneck.

This study delves into a multifaceted approach to address the scarcity of annotated lunar surface data. By employing a combination of machine learning and deep learning techniques, data augmentation, and strategic annotation efforts, we aim to optimize model performance and enhance the accuracy of lunar crater identification.

Table 7 highlights the average recall of subsets, showcasing the model's performance. Table 8 illustrates the average precision of subsets in the proposed lunar crater detection model. Meanwhile, Table 9 provides insights into the model's performance through F1 score and accuracy, complementing the already obtained average recall and precision values for subsets. In this analysis, the achieved accuracy is 86.91, accompanied by an impressive F1 measure of 0.7958.

**Table 7:** Performance of the model in terms of Average Recall (AR) lunar crater detection (with annotation).

Subset no	IOU threshold	Area	Max detections	Average recall
1	0.50:0.95	All	1	0.55
2	0.5	All	10	0.75
3	0.75	All	100	0.84
4	0.50:0.95	Small	100	0.87
5	0.50:0.95	Large	100	0.78
6	0.50:0.95	All	100	0.89
7	0.50:0.95	All	1	0.59
8	0.5	All	10	0.69
9	0.75	All	100	0.79
10	0.50:0.95	Small	100	0.86
11	0.50:0.95	Large	100	0.80
12	0.50:0.95	Medium	100	0.78
13	0.50:0.95	All	1	0.69
14	0.5	All	10	0.79
15	0.75	All	100	0.64
16	0.50:0.95	Small	100	0.67
17	0.50:0.95	Large	100	0.87
18	0.50:0.95	Medium	100	0.87
19	0.50:0.95	All	10	0.43
20	0.5	All	100	0.52
21	0.75	Small	100	0.69
22	0.50:0.95	Large	100	0.66
23	0.50:0.95	Medium	100	0.79
24	0.50:0.95	All	1	0.62

**Table 8:** Performance of the model in terms of average precision (AP) lunar crater detection (with annotation.

Subset no	IOU threshold	Area	Max detections	Average precision
1	0.50:0.95	All	1	0.59
2	0.5	All	10	0.63
3	0.75	All	100	0.69
4	0.50:0.95	Small	100	0.70
5	0.50:0.95	Large	100	0.68
6	0.50:0.95	All	100	0.71
7	0.50:0.95	All	1	0.51
8	0.5	All	10	0.59
9	0.75	All	100	0.63
10	0.50:0.95	Small	100	0.69
11	0.50:0.95	Large	100	0.70
12	0.50:0.95	Medium	100	0.66
13	0.50:0.95	All	1	0.59
14	0.5	All	10	0.60
15	0.75	All	100	0.56
16	0.50:0.95	Small	100	0.59
17	0.50:0.95	Large	100	0.69
18	0.50:0.95	Medium	100	0.60
19	0.50:0.95	All	10	0.59
20	0.5	All	100	0.59
21	0.75	Small	100	0.56
22	0.50:0.95	Large	100	0.57
23	0.50:0.95	Medium	100	0.59
24	0.50:0.95	All	1	0.512

**Table 9:** Performance of model based on F1-score and accuracy for annotated data.

Subset no	F1 score	Accuracy(%)
1	0.5698	71.39
2	0.6884	76.47
3	0.7596	83.49
4	0.7775	84.57
5	0.7321	82.64
6	0.7958	86.91
7	0.5509	61.71
8	0.6369	71.39
9	0.7016	76.35
10	0.7695	83.85
11	0.7539	85.42
12	0.7208	80.46
13	0.6432	72.35
14	0.6895	73.44
15	0.6037	68.84
16	0.6305	71.39
17	0.7700	83.49
18	0.7138	72.72
19	0.5029	72.35
20	0.5591	72.55
21	0.6224	68.6
22	0.6203	70.05
23	0.6784	71.39
24	0.5620	61.95

**Figure 11:**
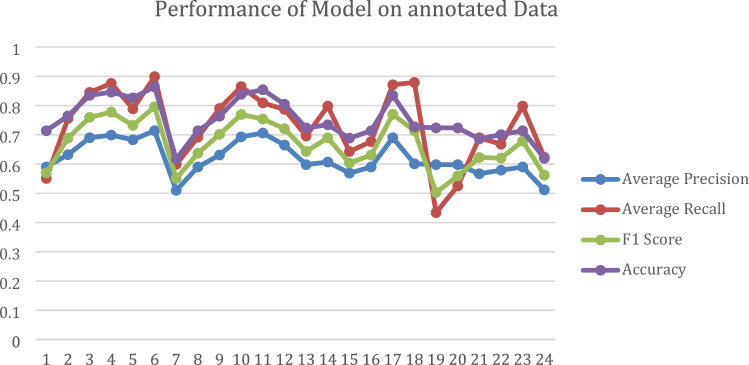
The Line graph displays the performance of the proposed model using annotated data.

Figure 11 is a line graph depicting the performance of the proposed model using unrelated data. This graphical representation includes average precision, recall, F1 score, and accuracy across all epochs, with values ranging from zero to one.

Some sample images of Chandrayan 2 TMC-2 images after data annotation are given in Table 10. This table also illustrates the different types of craters annotated.

**Table 10:**
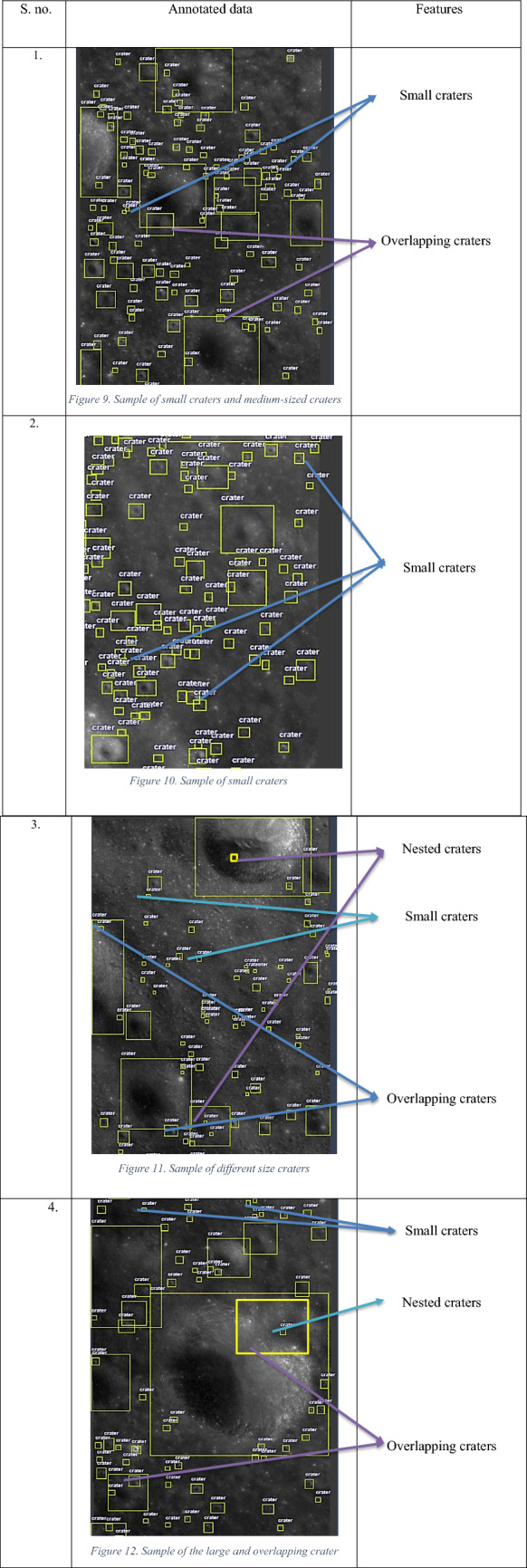
Annotated data samples.

### Predictions by the proposed model

The proposed crater prediction model is now trained on annotated data taken from Terrain Mapping Camera-2 (TMC-2) images of Chandrayaan-2 to explore lunar craters. The model trained using annotated data provided predictions of craters on the lunar images. Some test results are provided in Table 11 of the original images referred to in Figs. 2 and 3. The Prediction column has a yellow box which is the prediction of lunar craters performed by the model. The model’s findings pave the door for a better understanding of the impact mechanisms that shaped the lunar surface.

**Table 11:**
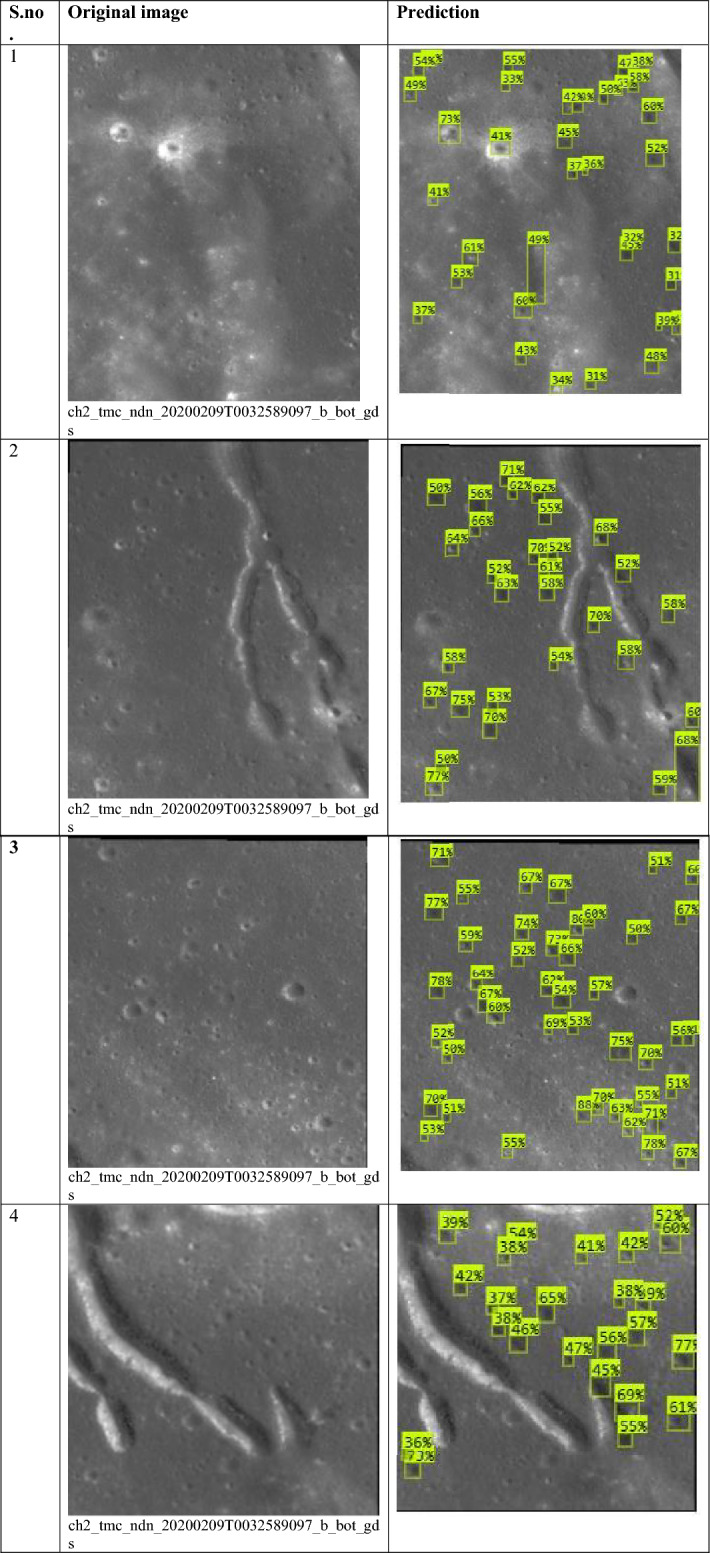
Prediction result is given by the model using TMC-2 images.

## Discussion

There are several measures available for gauging the performance of a trained neural network, these metrics might not be the best ones to use for determining how well a model identifies craters in a particular area. Scientists are primarily interested in whether the craters are correctly detected, rather than individual pixel-level accuracy. Therefore, it is more relevant to derive a metric based on the actual number of detected craters. This is done using the F1 score, which calculates the harmonic mean of recall and accuracy for each observed crater. To get the F1 score for crater counts, we must first define TP (true positives), FN (false negatives), and FP (false positives). The number of craters that the pipeline correctly identified and matched the human-annotated set is expressed as TP. The number of craters from the human annotation list that the pipeline is unable to locate is represented by the letter FN. The number of craters that the pipeline identifies but does not match the human annotation list is the difference between the number of identified craters and the number of matches, or FP. Recall is the ratio of true positives to the sum of true positives and false positives in a binary classification job, whereas accuracy is the ratio of true positives to the sum of true positives and false negatives. The average precision value of overall potential recall levels is known as average precision (AP).

**Figure 12:**
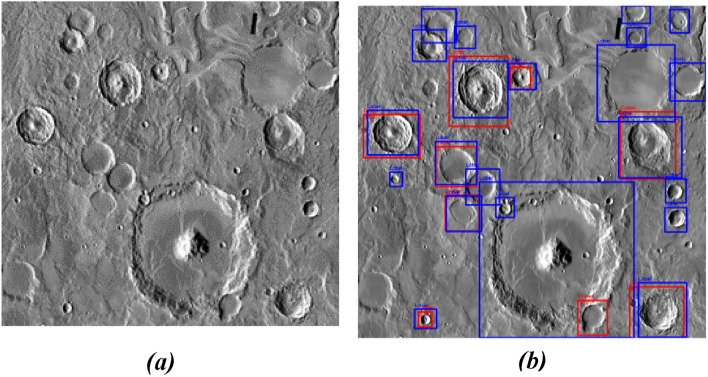
(**a**) Training image, and (**b**) Test Image 2 (red: labeled, blue: predicted).

Figure 12a is an image from the Mars_Lunar dataset used for training the proposed model. Figure 12b is the tested image in which the red-boxed craters are labeled and the blue ones are predicted.

*Precision* The fraction of detected craters that are true positives, i.e., correctly identified as craters.

$$Precision=\frac{TP}{TP+FP}$$

*Mean Precision* The average of the precision values for each class or label. It is calculated as the sum of the precision values for each class divided by the number of classes.

*Recall* The fraction of true craters that are detected, i.e., the sensitivity of the detection method.

$$Recall=\frac{TP}{TP+FN}$$

*F1-score* The harmonic mean of precision and recall, i.e., a measure of the overall accuracy of the detection method.

$$F1=2\times \frac{precision\times recall}{precision+recall}$$

*Accuracy* The number of correctly classified crater instances over the total number of crater instances.

$$Accuracy=\frac{TN+TP}{TN+FP+FN+TP}$$

The precision-recall curve, which shows precision as a function of recall, is summarized by the AP score. When all positive samples are correctly ordered at the top of the list, an AP score with a maximum value of 1 denotes greater results. The COCO evaluation metrics which is used to evaluate the object detections. The evaluation is predicated on the Intersection over Union (IoU) between the expected bounding boxes and the ground truth bounding boxes for the objects in the images.

Training for each epoch took approx 600 s while running on a Tesla T4 GPU, while the inference of each image takes about 800 ms.

### Performance on unannotated data

The model’s accuracy (Table 5) of 80.95% and the F1 measure at 0.7519 demonstrated admirable performance against unannotated data. The model has a high degree of accuracy and recall when it comes to identifying lunar craters. A balanced trade-off of precision against recall, necessary for successful crater detection, is suggested by the competitive F1 Measure. The model's ability to generalize well and recognize patterns inherent to lunar craters is highlighted by its success in unannotated data. The absence of annotation of TMC 2 in the training data significantly impacted the model's ability to learn and generalize effectively.

### Performance on annotated data

The model demonstrated even better performance when evaluated on a dataset of two thousand annotated images. The F1 Measure ascended to 0.79589% and the accuracy to 86.91% (Table 9). According to these results, the model gains significant benefits from training on labeled data since it can change its parameters and become more successful at differentiating between the crater and non-crater characteristics. The increased accuracy and F1 Measure on the annotated data indicate that the model might benefit from the extra information that annotations provide to improve its crater detection performance. This is due to the generally accepted notion that supervised learning generally produces better results than unsupervised methods, especially when using a well-annotated dataset. It is often advantageous to incorporate annotated data to provide supervision and context for better model performance.

Model after deployment shows remarkable results as shown in Table 11. The prediction images show very little misclassification, and a greater number of craters are detected. Even Though our proposed Model has shown encouraging results, deep learning, and machine learning approaches demand constant learning requirements. So, lunar crater detection approaches will continue and require dedicated contributions for data annotation, improvement of the model, and integration of advanced techniques.

The best-performing subset was Subset No. 6 which was across a range of IOU thresholds (0.50–0.95), considered on medium areas in the dataset, and allowed a maximum of a hundred detections per object. The average recall achieved was 0.87. On considering, large areas in the dataset, allowing a maximum of a hundred detections per object. The average precision achieved was 0.67. The F1-Score achieved was 0.7519 with the accuracy was 80.96% for the unannotated Chandrayaan-2 TMC-2 dataset.

The best-performing subset was Subset No. 6 which was across a range of IOU thresholds (0.50–0.95), considered on medium areas in the dataset, and allowed a maximum of a hundred detections per object. The average recall achieved was 0.89. On considering, large areas in the dataset, allowing a maximum of a hundred detections per object. The average precision achieved was 0.71. The F1-Score achieved was 0.7958 with the accuracy was 86.91% for annotated Chandrayaan-2 TMC-2 dataset.

The performance of the proposed model on unannotated and annotated data was according to our expectations as the performance of annotated data is more than that of unannotated data of TMC 2. We would further like to improve our accuracy by annotating more images and training the model with these images.

## Limitations

Even though our offers valuable insights into the prediction of lunar crater identification have some limitations. The size of the image title used in the dataset is 256 × 256 whereas the size of the original images from the TMC-2 sensor varies. The image size is important for the model performance because it affects the speed and accuracy of the object detection. Generally, smaller images are faster to process but may lose some details or features that are relevant for identifying the craters. Larger images are slower to process but may capture more details or features that are helpful for object detection. Therefore, choosing an appropriate image size is a trade-off between speed and accuracy. Currently, we have trained our model with only 2000 annotated images of the TMC-2 sensor from the Chadrayaan-2 satellite^30^. Further, in extended work, we strive to find an acceptable balance between processing efficiency and detection precision by carefully selecting image sizes. We also intend to use ArcGIS Pro with DEM images to improve crater detection accuracy. Furthermore, an approach will be built involving slicing images into numerous tiles of varied sizes, followed by annotation, to correct the model’s bias toward recognizing smaller craters. To assess our suggested methodology and investigate different competing approaches like YOLO, RCNN, and U-Net++, we will conduct extensive training, testing, and validation to design our model more robust to detect large as well as very small craters. To reduce the time consumption of the annotation procedure, we want to develop a qualitative heuristic equation for automatic bordering box generation. Our ultimate objective is to establish a reliable crater identification model by comparing different approaches. Despite obtaining promising accuracy rates of 80.95% (unannotated data) and 86.91% (annotated data), we are still devoted to improving our model's ability to identify a wider range of craters.

## Conclusion

Our work presents a novel approach for automated lunar crater detection that employs a bespoke semantic segmentation model based on U-Net with a ResNet50 backbone. For effective training, the model combines a pre-trained ResNet18 on ImageNet and is applied to TMC-2 ortho images from the Chandrayaan-2 satellite. The model, which was trained on a heterogeneous dataset that included Martian and Lunar crater images from multiple sources, makes use of YOLOv5 and Roboflow for data annotation and preprocessing. On unannotated data, the model performs well, achieving 80.95% accuracy and a 0.7519 F1 measure. The accuracy rises to 86.91% on an annotated dataset, with a 0.79589 F1 measure, demonstrating the model's capacity to generalize from labeled data. Understanding challenges like the requirement for better image annotation, the model's accuracy can be improved particularly in recognizing untrained crater portions by applying new tactics such as YOLO v7, YOLO v5, point-based detection approaches, Faster RCNN, and U-Net++. The presented model offers promising results for automating lunar crater detection, contributing valuable insights to lunar exploration and scientific research, and can be further used as an input for the Lunar surface age detection algorithm. The performance of the proposed model can be further improved by using TMC-2 DEM images and Chandrayaan-2 OHRC images. Annotation will be performed in ArcGIS software for accurately locating the craters in DEM images. Further in the study, using the data available from Mangalyaan and Chandrayaan- 2, training will be done on the same model as well and different models will also be tested. This will also be done to mitigate over-fitting challenges. As well as to cover the study area i.e. Vallis Schroter which focuses on locating tiles representing the Largest Rille on the Moon.

## Data Availability

We get the data from Pradan, ISRO https://pradan.issdc.gov.in/ch2/.
